# Synergistic Ternary Carbon Composite for Enhanced Simultaneous Electrochemical Sensing of Ascorbic Acid, Dopamine, and Uric Acid

**DOI:** 10.3390/mi17050588

**Published:** 2026-05-11

**Authors:** Yu-Ching Weng, Chen-Yu Wu

**Affiliations:** Department of Chemical Engineering, Feng Chia University, Taichung 407102, Taiwan; d1125101@o365.fcu.edu.tw

**Keywords:** graphene, carbon nanotube, XC72, ascorbic acid, dopamine, uric acid, electrochemical sensor

## Abstract

Simultaneous quantification of ascorbic acid, dopamine, and uric acid is crucial for clinical diagnostics. Here, an electrochemical sensor was developed by modifying a glassy carbon electrode with a ternary composite of multi-walled carbon nanotubes, graphene, and Vulcan XC72 carbon black via a simple mixing method. The synergistic interaction of these carbon materials significantly increases the electroactive surface area and introduces defect-driven catalytic sites, enhancing electron transfer kinetics. The sensor enables interference-free simultaneous detection, exhibiting linear ranges of 100–1000 μM ascorbic acid, 5–50 μM dopamine, and 10–100 μM uric acid with sensitivities of 0.044, 0.47, and 0.95 μA μM^−1^, respectively, and corresponding limits of detection of 34.1, 4.23, and 11.1 μM. The platform also demonstrated excellent stability, reproducibility, and anti-interference performance, with satisfactory recoveries in human urine samples. These results highlight the ternary composite sensor as a reliable and practical tool for multiplexed monitoring in complex physiological matrices.

## 1. Introduction

Global aging drives the demand for sensitive biosensors capable of simultaneous multi-analyte detection. In human physiological metabolism, concentration fluctuations of ascorbic acid (AA), dopamine (DA), and uric acid (UA) are closely linked to various major pathologies, including scurvy, depression, and chronic kidney disease; thus, the timely and accurate monitoring of these species is of paramount importance for disease prevention and clinical diagnosis. AA, or Vitamin C, is a well-recognized potent antioxidant and free radical scavenger [1]. Due to its antioxidant properties, AA is widely employed in the prevention and treatment of conditions such as the common cold, scurvy, psychiatric disorders, infertility, cancer, and AIDS [2]. Given its multifaceted biological roles, monitoring AA is clinically significant, with normal human serum concentrations typically ranging from 23 to 85 μM [3]. DA, a vital catecholamine neurotransmitter primarily localized within the central nervous system [4], plays a pivotal role in motor control, emotional regulation, and cognitive functions such as learning and memory. While its physiological concentration in serum is relatively low (0.01–1.0 μM) [3], abnormal DA levels are frequently implicated in neurological disorders such as schizophrenia and Huntington’s disease [5], as well as mood disorders, substance addiction, and diverse metabolic diseases [6]. Furthermore, UA serves as the primary end-product of purine metabolism, maintaining a physiological serum range between 214 and 494 μM [3,7]. Deviations in UA levels are critical diagnostic indicators for Lesch–Nyhan syndrome, gout, and pneumonia [8]. Specifically, the crystallization of UA in joints can trigger painful inflammatory responses leading to gout [9], while its excessive accumulation may result in renal calculi and subsequent impairment of kidney function. Thus, the precise detection of these three biomarkers is essential for the early diagnosis of various neurological and metabolic diseases.

Commonly used biomolecule detection methods currently include capillary electrophoresis [10], colorimetry [11], and spectrophotometry [12], yet these techniques generally face limitations such as high costs, time-consuming procedures, and complex sample pre-treatment; in contrast, electrochemical methods [13,14], as a significant branch of chemical sensors, operate by measuring electrical signal variations—such as potential, current, or conductivity—during the reaction process to convert these changes into analyte concentrations [15,16], leveraging their advantages in terms of high cost-effectiveness, operational simplicity, superior sensitivity, and favorable limits of detection (LOD) to enable the precise quantification of trace analytes within biological fluids [17]. However, AA, DA, and UA, as electroactive species, often exhibit overlapping oxidation potentials and are susceptible to mutual interference due to their similar electrochemical properties [18]. Existing research indicates that electrode modification with carbon-based nanomaterials and the incorporation of metal nanoparticles can effectively resolve and sense these three substances [19]. Sun et al. [20] employed an electrodeposition technique to decorate molybdenum disulfide (MoS_2_) nanosheets with gold nanoparticles (AuNPs), achieving high-sensitivity simultaneous detection of AA, DA, and UA through superior electrocatalytic activity and significant peak potential separation. Meissam Noroozifar et al. [21] developed a glassy carbon electrode modified with iron ion-doped natrolite zeolite (Fe-NZ) and multiwalled carbon nanotubes (MWCNTs), achieving high-sensitivity simultaneous detection of AA, DA, UA, and Trp through significant peak potential separation and micromolar-level detection limits. Nevertheless, some fabrication processes for these specialized materials are characterized by complexity, time-consuming protocols, and high production costs.

Given that carbon nanomaterials have been extensively utilized in sensor development, they have also been successfully employed for the electrochemical detection of AA, DA, and UA [22]. Zhang et al. [3] utilized a direct thermal oxidation method to synthesize porous graphitic carbon nitride (PCN) nanosheets composited with graphene oxide (GO) to construct a sensor with high specific surface area and exceptional electrocatalytic activity for the simultaneous determination of AA, DA, and UA. Wang et al. [8] developed a glassy carbon electrode (GCE) modified with carbon black (CB) and carbon nanotube (CNT) co-doped polyimide (PI) to achieve the simultaneous detection of AA, DA, and UA in human urine samples. Drawing upon established precedents in the literature, a composite consisting of MWCNTs, GR, and Vulcan XC-72 was selected as the modifier for the development of the electrochemical sensor. CNTs consist of hollow cylindrical structures formed by rolled graphene layers with sp^2^-hybridized carbon networks. In MWCNTs, the tube walls are composed of conjugated π-electron systems, which endow them with high chemical stability, large specific surface area, and excellent electron transfer capability [14]. Furthermore, compared to single-walled carbon nanotubes (SWCNTs), MWCNTs are generally easier to synthesize and involve less complex preparation procedures, making them more cost-effective for large-scale applications. However, strong van der Waals interactions between MWCNTs often lead to severe aggregation, thereby limiting their electrical conductivity [23,24]. GR, also composed of sp^2^-hybridized carbon atoms, features a two-dimensional honeycomb lattice structure with in-plane σ bonds and out-of-plane π bonds. This unique configuration provides exceptional mechanical strength, high electrical conductivity, and a large surface area [25]. Moreover, the superior conductivity of GR can compensate for the conductivity loss of MWCNTs caused by aggregation. CB, such as Vulcan XC-72, possesses a highly mesoporous structure, good electrical properties, facile preparation, and low cost [26,27].

Despite the promising properties of individual carbon nanomaterials, their practical application in electrochemical sensing is often hindered by structural limitations, such as graphene restacking and carbon nanotube aggregation. To address these challenges, the rational design of hybrid architectures with complementary dimensional features is essential. In this study, we propose a three-dimensional (3D) hierarchical carbon nanocomposite by integrating zero-dimensional (0D) Vulcan XC-72, one-dimensional (1D) MWCNTs, and two-dimensional (2D) GR. The incorporation of XC-72 as a nanoscale spacer effectively mitigates the aggregation of both MWCNTs and GR, leading to the formation of a point–line–plane conductive network with enhanced structural stability and dispersibility. This unique architecture not only increases the electrochemically active surface area but also facilitates rapid electron transfer and improves mass transport.

## 2. Materials and Methods

### 2.1. Materials

Ascorbic acid (C_6_H_8_O_6_, 99%) was obtained from Tokyo Chemical Industry (Tokyo, Japan). Dopamine hydrochloride (C_8_H_12_ClNO_2_, 99%), uric acid (C_5_H_4_N_4_O_3_, 99%), L-fucose (C_6_H_12_O_5_, 97%), and creatinine (C_4_H_7_N_3_O, 99%) were purchased from Thermo Fisher Scientific (Waltham, MA, USA). Multiwalled carbon nanotubes (MWCNTs) were supplied by Taiwan Carbon Materials Corp. (Taoyuan, Taiwan), while graphene (GR) was purchased from Strem (Newburyport, MA, USA). Vulcan XC-72 carbon black was obtained from Cabot Corp. (Boston, MA, USA) N,N-dimethylformamide (DMF, C_3_H_7_NO, 99.5%) was purchased from J.T. Baker (Radnor, PA, USA). Potassium dihydrogen phosphate (KH_2_PO_4_, 99.5%), disodium hydrogen phosphate (Na_2_HPO_4_, 99%), potassium chloride (KCl, 99.5%), glucose (C_6_H_12_O_6_, 98%), and sodium sulfate (Na_2_SO_4_, 99%) were obtained from SHOWA (Tokyo, Japan). Sodium chloride (NaCl, 99%) was purchased from Fisher Chemical (Waltham, MA, USA). Hydrochloric acid (HCl, 37%) and sodium hydroxide (NaOH) were obtained from Honeywell (Charlotte, NC, USA). For electrochemical measurements, a platinum (Pt) electrode (0.5 × 0.5 cm^2^) was purchased from Leesan Precious Metal Co., Ltd. (Tainan, Taiwan), and a glassy carbon electrode (GCE, 3 mm diameter) was obtained from Aurora (Taipei, Taiwan). The Ag/AgCl (3.5 M KCl) reference electrode was fabricated in-house.

### 2.2. Preparation of MWCNT-GR-XC72/GCE

All chemicals used in this study were of analytical grade and used as received without further purification. A homogeneous suspension was prepared by dispersing 0.3 wt% MWCNTs, 0.1 wt% GR, and 0.1 wt% XC-72 in DMF, followed by ultrasonication for 30 min and magnetic stirring for at least 40 min. The GCEs were sequentially polished with alumina slurries of 0.5 and 0.05 μm, and then ultrasonically cleaned in deionized water for 10 min to remove residual contaminants. Subsequently, 1 μL of the prepared MWCNT–GR–XC72 suspension was drop-cast onto the cleaned GCE surface and dried in an oven for 5 min. This procedure was repeated twice to achieve a total loading volume of 2 μL, yielding the MWCNT–GR–XC72/GCE.

### 2.3. Material Characterization and Electrochemical Measurements

The surface morphology of the MWCNT–GR–XC72 composites was characterized by transmission electron microscopy (TEM, JEM-2100Plus, JEOL, Tokyo, Japan). The crystalline structure of the composite materials was analyzed using high-resolution X-ray diffraction (XRD, D8 Discover, Bruker, Billerica, MA, USA). The structural defects and graphitization degree of the composites were investigated by Raman spectroscopy (AvaSpec-ULS2048LTEC-USB2, AVANTES, Apeldoorn, The Netherlands). Electrochemical measurements were performed using SP-240 (BioLogic, Seyssinet-Pariset, France) and CHI 900D (CH Instruments, Austin, TX, USA) potentiostats. A conventional three-electrode system was employed, consisting of MWCNT–GR/GCE or MWCNT–GR–XC72/GCE as the working electrode, a platinum wire as the counter electrode, and an Ag/AgCl (3.5 M KCl) electrode as the reference electrode. All experiments were conducted in 0.1 M phosphate-buffered saline (PBS) as the supporting electrolyte. Cyclic voltammetry (CV) measurements for the simultaneous detection of AA, DA, and UA were performed over a potential range of −0.2 to 0.6 V (vs. Ag/AgCl) at a scan rate of 50 mV s^−1^. For quantitative analysis with enhanced sensitivity and lower detection limits, differential pulse voltammetry (DPV) was employed. DPV measurements were conducted over the same potential range (−0.2 to 0.6 V), with a pulse amplitude of 0.05 V, a pulse width of 0.1 s, and a pulse period of 0.5 s. Unless otherwise specified (e.g., continuous measurements), all experiments were carried out using freshly prepared electrodes.

## 3. Results and Discussion

### 3.1. Characterization of MWCNT-GR-XC72/GCE

**Figure 1:**
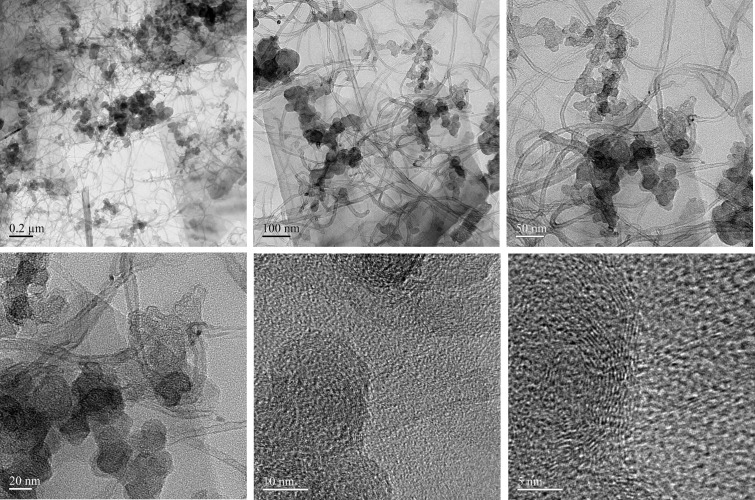
TEM images of the MWCNT–GR–XC72 composite at different magnification.

The morphology and spatial distribution of the MWCNT–GR–XC72 ternary carbon composite (mass ratio of 3:1:1) were characterized by TEM at various magnifications, as shown in Figure 1. The TEM images reveal that GR exhibits a large-area, ultrathin, lamellar structure, whereas MWCNTs form fibrous, hollow tubular networks with an average diameter of approximately 5 nm. The dark spherical particles are attributed to XC-72, which inherently exhibits a certain degree of agglomeration, with an average particle diameter of approximately 30 nm. Notably, the TEM results indicate that the incorporation of XC-72 effectively suppresses the restacking of GR sheets and improves the overall dispersion of the composite. Consequently, the ternary carbon structure exhibits enhanced dispersibility, leading to an increased electroactive surface area. Furthermore, the synergistic integration of the three carbon materials facilitates improved electrical conductivity, benefiting from their complementary structural characteristics and defect-rich features.

**Figure 2:**
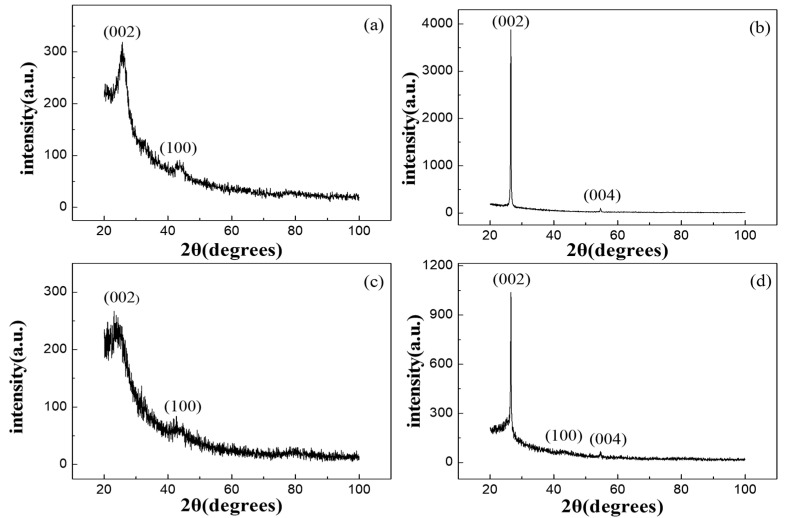
XRD patterns of (**a**) MWCNT, (**b**) GR, (**c**) XC72, and (**d**) MWCNT-GR-XC72.

Following the morphological characterization of the ternary carbon composites via TEM, XRD analysis was employed to elucidate the crystallinity and crystallographic planes of the three constituent carbon materials and their resulting composite. Figure 2a presents the XRD pattern of the MWCNT, where the diffraction peaks at 25.74° and 43.65° correspond to the (002) and (100) planes, respectively. These values are in excellent agreement with the hexagonal graphite phase (JCPDS 41-1487) [28]. The XRD pattern of GR is shown in Figure 2b, exhibiting two characteristic peaks at 26.5° and 54.6°, assigned to the (002) and (004) planes of hexagonal graphite (JCPDS 41-1487) [29,30]. The intensity and position of these peaks confirm the existence of a highly graphitized state and well-preserved structural integrity. In contrast, the XRD pattern of XC72 (Figure 2c) displays diffraction peaks at 24.11° and 42.36°, corresponding to the (002) and (100) planes (JCPDS 41-1487) [31,32,33]. However, these peaks are notably broad and low in intensity, which is characteristic of a typical amorphous carbon structure. Finally, the XRD pattern of the integrated MWCNT-GR-XC72 composite is illustrated in Figure 2d. The peaks observed at 26.18°, 42.96°, and 54.43° correspond to the (002), (100), and (004) planes of hexagonal graphite. Notably, the composite retains the intense (002) signal derived from the graphene, while also incorporating the broader base features characteristic of XC72 and MWCNTs. This confirms that the composite successfully preserves both the highly conductive crystalline framework of graphene and the defect-rich, high-surface-area structure provided by XC72. Consequently, the XRD results in Figure 2d substantiate that the proposed simple mixing process ensures excellent dispersion and the successful fabrication of the ternary carbon nanomaterial composite.

**Figure 3:**
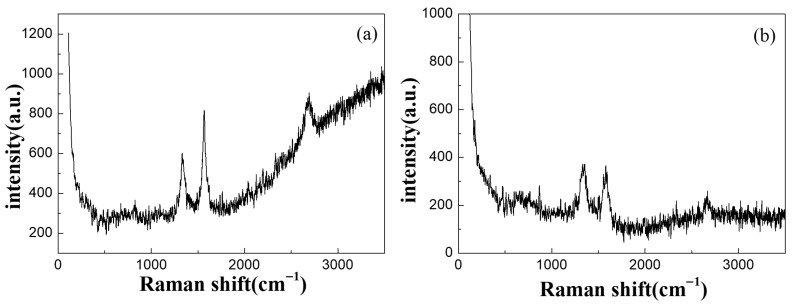
Raman spectra of (**a**) MWCNT-GR and (**b**) MWCNT-GR-XC72.

Complementing the morphological insights from TEM and the crystal plane identification through XRD, Raman spectra further verify that the introduction of XC72 effectively introduces additional defect sites into the composite. Figure 3a,b illustrate the Raman spectra of the MWCNT-GR and MWCNT-GR-XC72 carbon composites. For MWCNT-GR, three characteristic peaks are observed at 1331, 1558, and 2686 cm^−1^, which are assigned to the D band (structural defects), G band (graphitic structure), and G′ band (second-order overtone of the D band), respectively [28]. Upon the incorporation of XC72, the composite exhibits similar characteristic peaks at 1336, 1579, and 2666 cm^−1^. It is well established that the relative intensity ratio of the D band to the G band (ID/IG) reflects the density of structural defects and the degree of sidewall functionalization [29]. As shown in Figure 3a,b, the ID/IG value increases from 0.73 for MWCNT-GR to 1.01 for MWCNT-GR-XC72. This enhancement indicates that the introduction of XC72 effectively increases the number of structural defects and induces sidewall functionalization, thereby providing more active sites and improving the electrical conductivity of the modified composite.

### 3.2. Effective Area of MWCNT-GR/GCE and MWCNT-GR-XC72/GCE

**Figure 4:**
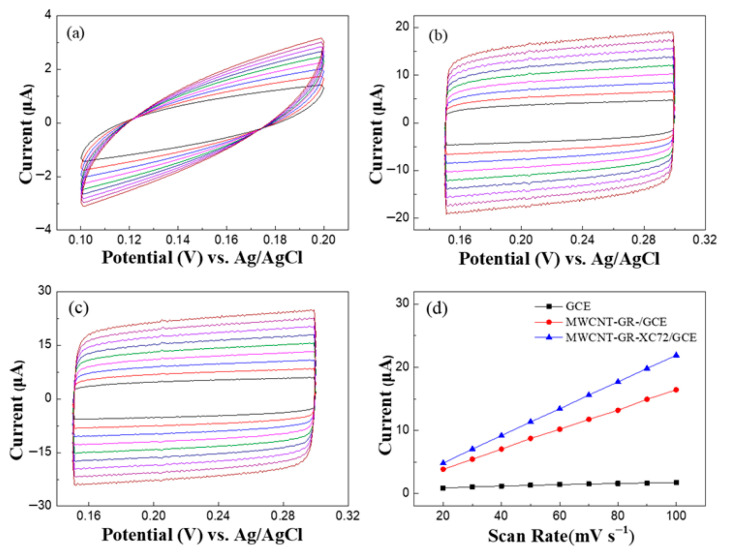
CVs of (**a**) bare GCE, (**b**) MWCNT-GR/GCE, and (**c**) MWCNT-GR-XC72/GCE with different scan rate (20~100 mVs^−1^) in 0.1 M PBS. (**d**) Peak current as a function of square root of scan rate for bare GCE (black), MWCNT-GR/GCE (red), MWCNT-GR-XC72/GCE (blue).

The electrochemical active surface area (ECSA) is typically determined through double-layer capacitance (C_DL_) measurements, providing a quantitative metric to evaluate whether the MWCNT-GR-XC72 ternary carbon composite effectively enhances the electroactive area of the modified electrode. Generally, the ECSA value is positively correlated with the magnitude of the electrochemical current response. To determine this, scan rate-dependent CV was conducted in a 0.1 M PBS solution (pH 7.0) in the absence of any analyte, covering a scan rate range from 20 to 100 mV/s, as shown in Figure 4a–c. By plotting the charging current (i_c_) as a function of the scan rate (*v*), as shown in Figure 4d, the C_DL_ of the material can be extracted from the resulting slope according to Equation (1) [34]:

(1) i_c_ = *v* C_DL_

(2) C_S_ = C_DL_/A

(3) ECSA = C_DL_/C_S_

The ECSA of the modified electrodes was evaluated based on the double-layer capacitance (C_DL_) measurements. By substituting the geometric area (A) of the bare GCE = 0.0707 cm^2^ into Equation (2), the specific capacitance C_S_ of the bare electrode was determined to be 0.1515 μF/cm^2^. Subsequently, the C_DL_ values for the MWCNT-GR/GCE and MWCNT-GR-XC72/GCE were calculated using Equation (1) and substituted into Equation (3) to derive their respective ECSA values. The results indicate that the ECSA of MWCNT-GR/GCE is 1.035 cm^2^, which is approximately 14.64 times greater than that of the bare GCE. Notably, the MWCNT-GR-XC72/GCE exhibited a higher ECSA of 1.406 cm^2^, representing a 19.88-fold increase relative to the GCE. These findings confirm that the incorporation of XC72 effectively enhances the electrochemically active surface area and plays a critical role in improving the dispersion of GR within the composite matrix.

### 3.3. Electrochemical Behavior Toward Oxidation of AA, DA, and UA

**Figure 5:**
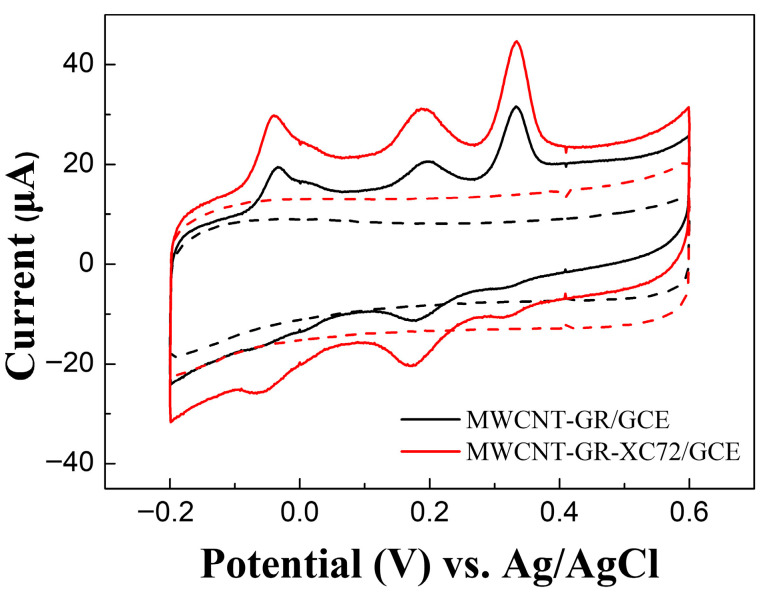
CVs of MWCNT–GR/GCE (black) and MWCNT–GR–XC72/GCE (red) recorded in 0.1 M PBS (pH 7.0) in the presence (solid lines) and absence (dashed lines) of 1000 μM AA, 50 μM DA, and 100 μM UA.

CV was employed to evaluate the sensing performance of the modified electrodes by recording redox currents over a defined potential window at a constant scan rate. Figure 5 presents the CV responses of MWCNT–GR/GCE and MWCNT–GR–XC72/GCE in 0.1 M PBS (pH 7.0) containing 1000 μM AA, 50 μM DA, and 100 μM UA, while the dashed curves represent the background signals in the absence of analytes. The voltammograms demonstrate that both modified electrodes enable the simultaneous detection of AA, DA, and UA, exhibiting well-resolved and non-overlapping oxidation peaks for each species. Notably, the MWCNT–GR–XC72/GCE shows significantly enhanced current responses compared to MWCNT–GR/GCE under both blank and analyte-containing conditions. The pronounced increase in oxidation peak currents upon the addition of target analytes indicates that the incorporation of XC-72 effectively improves the electrocatalytic activity and sensitivity of the composite electrode. Although the CV results qualitatively confirm the superior sensing performance of the XC-72-modified electrode, further quantitative evaluation was carried out using DPV to achieve higher sensitivity and resolution.

### 3.4. Influence of Scan Rate

**Figure 6:**
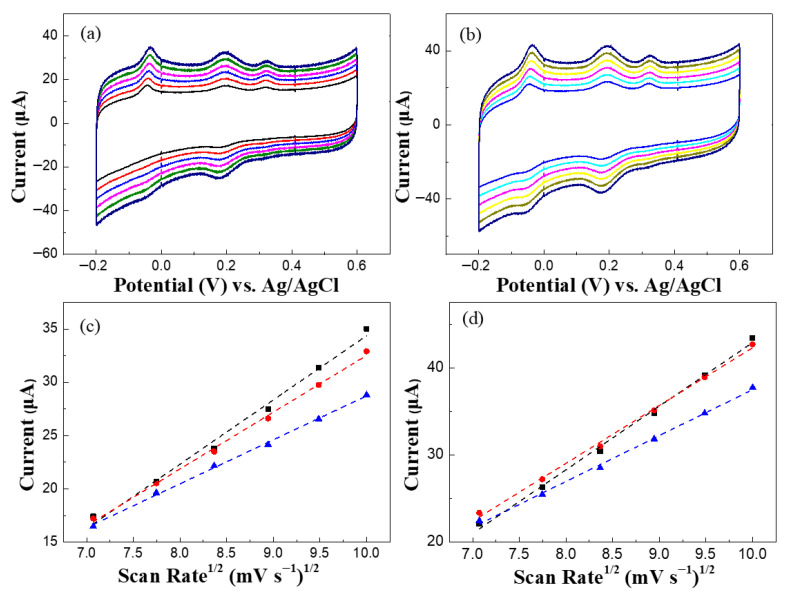
CVs of (**a**) MWCNT–GR/GCE and (**b**) MWCNT–GR–XC72/GCE recorded at scan rates from 50 to 100 mV s^−1^ in 0.1 M PBS containing 1000 μM AA, 50 μM DA, and 100 μM UA. Plots of anodic peak currents versus the square root of scan rate for (**c**) MWCNT–GR/GCE and (**d**) MWCNT–GR–XC72/GCE.

To evaluate the effect of scan rate on the modified electrodes, CV was conducted at scan rates ranging from 50 to 100 mV s^−1^. Figure 6a,b show the CV responses of MWCNT–GR/GCE and MWCNT–GR–XC72/GCE, respectively, for the simultaneous detection of 1000 μM AA, 50 μM DA, and 100 μM UA at different scan rates. It was observed that the oxidation peak currents (I_pa_) for all three analytes increased significantly with the increment of the scan rate. To further elucidate the mass transport mechanism, the relationship between the peak currents and the square root of the scan rate (*v*^1/2^) was analyzed, as shown in Figure 6c,d. The experimental data for both the MWCNT-GR/GCE and MWCNT-GR-XC72/GCE exhibited excellent linearity. These results confirm that the electrochemical redox reactions of AA, DA, and UA on both modified surfaces are primarily diffusion-controlled processes [35]. Furthermore, the data demonstrates that both electrodes maintain high efficacy for the simultaneous sensing of AA, DA, and UA within the investigated scan rate range of 50–100 mV/s.

### 3.5. Influence of pH and Reaction Mechanism

**Figure 7:**
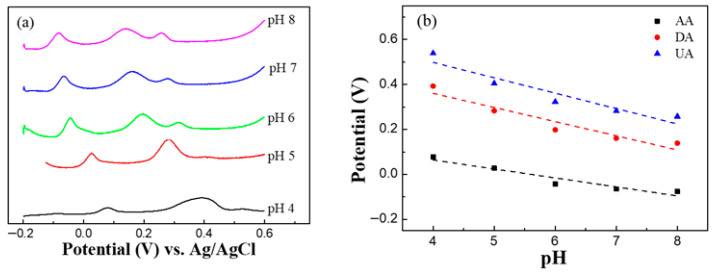
(**a**) CVs of MWCNT-GR-XC72/GCE in 0.1 M PBS containing 1000 μM AA, 50 μM DA, and 100 μM UA at various pH values. (**b**) Dependence of the oxidation peak potentials of AA, DA, and UA on solution pH.

**Scheme 1:**
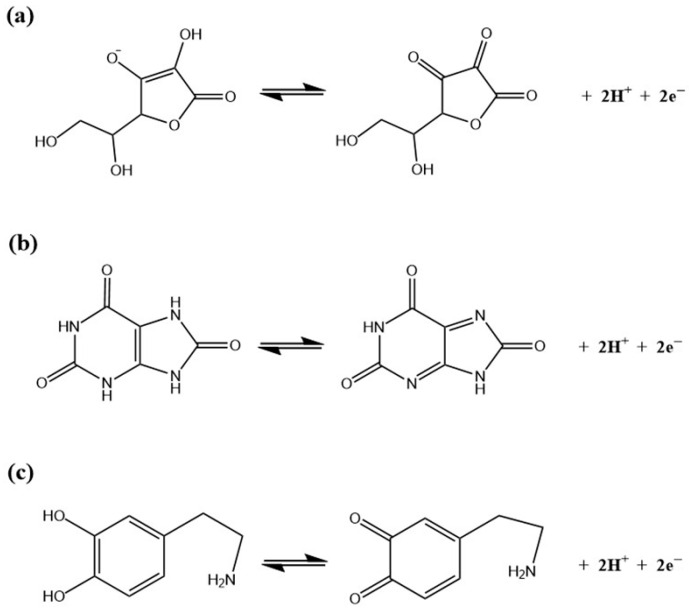
Proposed electrochemical redox mechanisms of (**a**) AA, (**b**) DA, and (**c**) UA.

By analyzing the potential shifts in AA, DA, and UA in solutions of varying pH, the electron and proton transfer phenomena during their respective oxidation processes can be investigated. Figure 7a illustrates the CV of the MWCNT-GR-XC72/GCE in response to AA, DA, and UA across a range of pH values. As the pH increased from 4.0 to 8.0, the oxidation peak potentials (E_pa_) for all three analytes shifted toward more negative values, indicating that protons are directly involved in the electrochemical redox reactions [36]. Figure 7b shows a negative linear correlation between the oxidation peak potential and the pH value. The corresponding linear regression equations were calculated as follows: AA: E = −0.0400 pH + 0.2242; DA: E = −0.0630 pH + 0.6128; UA: E = −0.0685 pH + 0.7724. The derived slopes (40.0, 63.0, and 68.5 mV/pH) are close to the theoretical Nernstian value of 59 mV/pH, suggesting that the number of protons and electrons transferred during the oxidation process is equal [37]. These results are consistent with the mechanisms proposed in Scheme 1, confirming that the oxidation of AA, DA, and UA involves a two-electron and two-proton transfer process.

### 3.6. DPVs for the Simultaneous Determination of AA, DA, and UA

**Figure 8:**
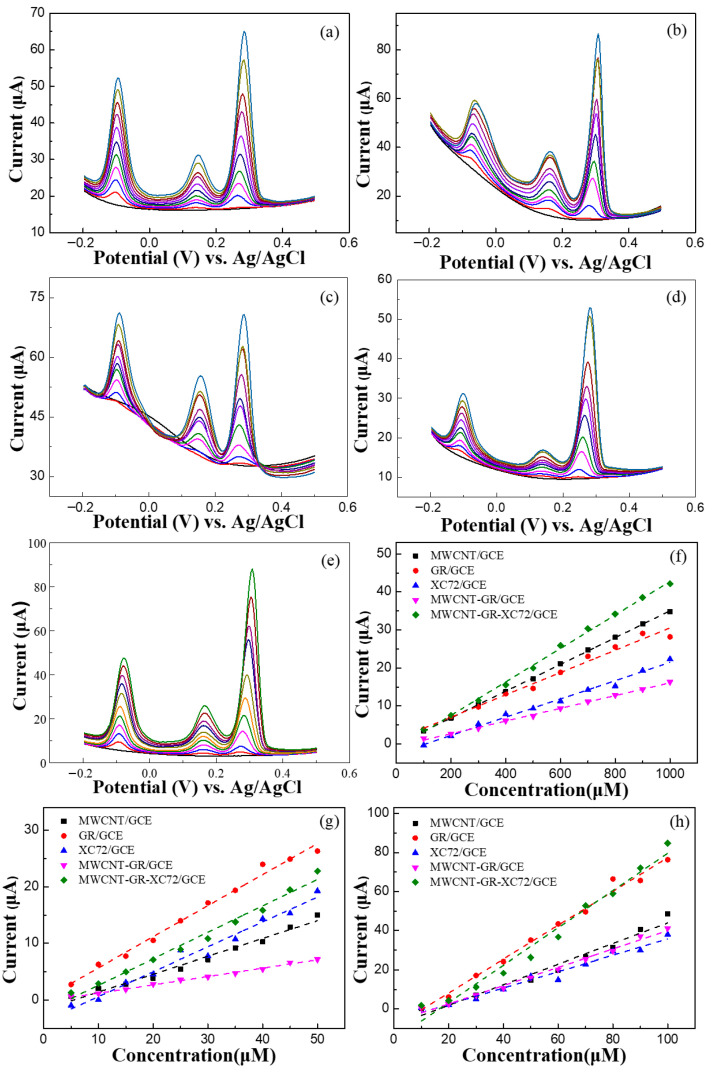
DPVs of (**a**) MWCNT/GCE, (**b**) GR/GCE, (**c**) XC72/GCE, (**d**) MWCNT-GR/GCE, and (**e**) MWCNT-GR-XC72/GCE in 0.1 M PBS containing various concentrations of AA (100–1000 μM), DA (5–50 μM), and UA (10–100 μM). The linear relationship between the peak current and concentration of (**f**) AA, (**g**) DA, and (**h**) UA on MWCNT-GR/GCE, and MWCNT-GR-XC72/GCE.

Following the confirmation of the simultaneous sensing capability via CV, DPV was employed for a more detailed quantitative analysis of the performance differences between the electrodes. Figure 8a–e display the DPV curves obtained for the MWCNT/GCE, GR/GCE, XC72/GCE, MWCNT-GR/GCE and MWCNT-GR-XC72/GCE in a 0.1 M PBS solution with sequential additions of 100–1000 μM AA, 5–50 μM DA, and 10–100 μM UA. The DPV parameters were configured with a potential scan range from −0.2 V to 0.6 V (vs. Ag/AgCl), a pulse amplitude of 0.05 V, a pulse width of 0.1 s, and a pulse period of 0.5 s. As illustrated in Figure 8, MWCNT/GCE, GR/GCE, XC72/GCE, MWCNT-GR/GCE, and MWCNT-GR-XC72/GCE all demonstrate the capability for the simultaneous and interference-free detection of AA, DA, and UA. Both MWCNT/GCE and GR/GCE exhibit superior peak resolution, which accounts for their frequent adoption in the prior literature for electrochemical sensor modification. Although XC72/GCE successfully resolves the three oxidation peaks, it suffers from significant baseline fluctuations. This instability is attributable to the poor adhesion of XC72 on the GCE surface, which prevents the formation of a robust carbon film. Notably, the MWCNT-GR/GCE and MWCNT-GR-XC72/GCE composites achieve the largest peak potential separations between DA and UA, indicating optimal sensing performance while effectively mitigating the stability issues associated with XC72. Figure 8f–h illustrate the relationship between the peak oxidation currents and the concentrations of AA, DA, and UA, designated as the concentration calibration curves. The results reveal a strong linear relationship between the response currents and the concentrations across the tested ranges (100–1000 μM AA, 5–50 μM DA, and 10–100 μM UA), where the slope of each calibration curve represents the sensitivity of the sensor. The limit of detection (LOD) was determined using the standard deviation of the response (σ) and the slope of the calibration curve (S) according to the IUPAC definition, Ref. [38] the calculation formulas are shown in Equation (4):

(4) LOD = 3.3 × σ/S

As summarized in Table 1, the sensitivities of single-component modified electrodes, such as MWCNT/GCE and XC72/GCE, for the simultaneous sensing of AA, DA, and UA are consistently lower than those achieved by the MWCNT-GR-XC72/GCE. Although GR/GCE exhibits the highest sensitivity toward DA, its performance for AA and UA detection is suboptimal; notably, its LOD for AA is significantly higher than that of other modified electrodes. Among all candidates, the MWCNT-GR-XC72/GCE demonstrates the most superior overall performance, with sensitivities of 0.044, 0.47, and 0.95 μA/μM for AA, DA, and UA, respectively. These values represent a 2.5-, 3.1-, and 2.0-fold increase compared to the binary MWCNT-GR/GCE. This enhancement is primarily attributed to the synergistic effects of the composite, specifically the high electrical conductivity, large specific surface area, and exceptional dispersibility of XC72 carbon black. These findings underscore the potential of MWCNT-GR-XC72/GCE for the simultaneous determination of these analytes in coexisting systems.

Additionally, Table 2 summarizes several glassy carbon electrodes modified with different materials, detailing their linear concentration ranges and LOD for the simultaneous sensing of AA, DA, and UA. Although the LOD of the proposed modified electrode may seem slightly less competitive compared to the previously reported ones—primarily because the LOD is inherently correlated with the boundaries of the analytical detection range—it nevertheless exhibits excellent sensitivity and an adequate LOD, particularly considering that the modification utilizes only three simple carbon materials.

### 3.7. Reproducibility, Repeatability, Stability and Selectivity of the Developed Sensor

**Figure 9:**
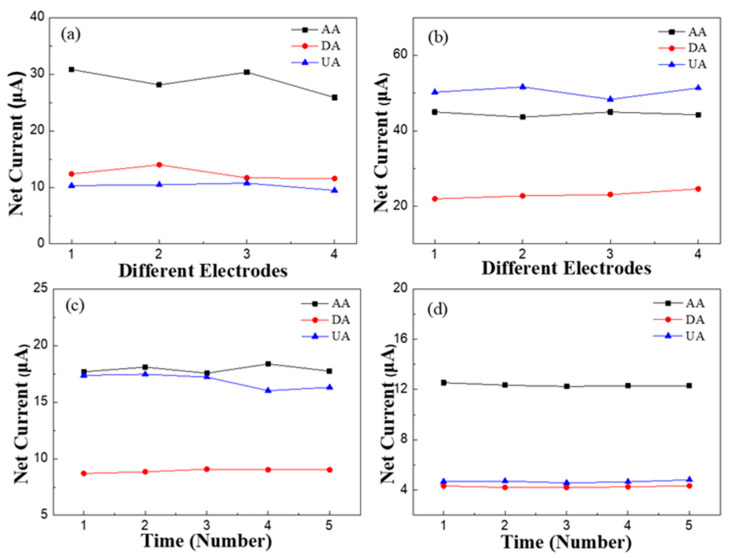
Net currents of (**a**) MWCNT-GR/GCE and (**b**) MWCNT-GR-XC72/GCE in 0.1 M PBS containing 1000 μM AA, 50 μM DA, and 100 μM UA for four independent electrodes. Net currents of (**c**) MWCNT-GR/GCE and (**d**) MWCNT-GR-XC72/GCE in the same solution over five consecutive measurements.

To evaluate the fabrication reproducibility of the proposed sensors, four independent MWCNT-GR/GCE and MWCNT-GR-XC72/GCE were prepared. Peak current variations were monitored using DPV, and the results are shown in Figure 9a,b. For MWCNT-GR/GCE, the relative standard deviations (RSDs) for AA, DA, and UA were 7.91%, 9.02%, and 5.47%, respectively. In contrast, MWCNT-GR-XC72/GCE exhibited significantly lower RSDs of 1.47%, 4.81%, and 2.95% for AA, DA, and UA, respectively. These results indicate that MWCNT-GR-XC72/GCE possesses superior fabrication reproducibility, with all RSDs well below the 5% threshold. Sensor repeatability was further evaluated through five consecutive DPV measurements using a single electrode under a fixed concentration, as shown in Figure 9c,d. The MWCNT-GR/GCE yielded RSDs of 1.88%, 1.73%, and 3.92% for AA, DA, and UA, respectively, whereas MWCNT-GR-XC72/GCE exhibited even higher precision, with RSDs of 0.92%, 1.45%, and 1.93% for the three analytes. The consistently low-repeatability RSDs for MWCNT-GR-XC72/GCE confirm the exceptional stability and reliable performance of this modified electrode for simultaneous electrochemical detection.

**Figure 10:**
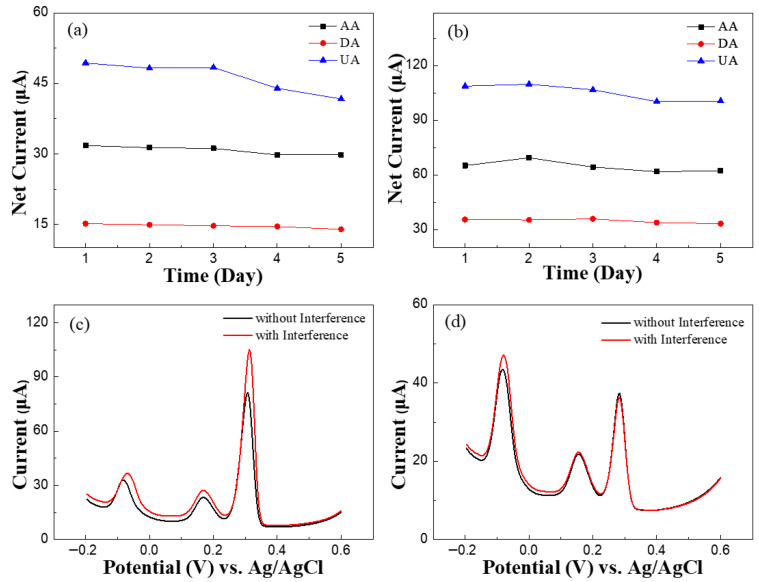
Net currents of (**a**) MWCNT-GR/GCE and (**b**) MWCNT-GR-XC72/GCE in 0.1 M PBS containing 1000 μM AA, 50 μM DA, and 100 μM UA over five consecutive days. Interference study of potential foreign substances in the presence of 500 μM AA, 30 μM DA, and 50 μM UA in 0.1 M PBS by DPV at (**c**) MWCNT-GR/GCE and (**d**) MWCNT-GR-XC72/GCE.

The stability of MWCNT-GR/GCE and MWCNT-GR-XC72/GCE was evaluated over five consecutive days, as shown in Figure 10a,b. The modified electrodes were stored under ambient conditions at room temperature, and daily DPV measurements were performed to monitor the retention of the net peak currents. After five days, MWCNT-GR/GCE retained 93.9%, 92.1%, and 84.5% of its initial current responses for AA, DA, and UA, respectively. In contrast, MWCNT-GR-XC72/GCE exhibited significantly higher stability, maintaining 95.7%, 93.7%, and 92.1% of its original signals. These results indicate that the incorporation of XC72 carbon black provides a more robust and stable sensing environment.

The selectivity of the modified electrodes was further investigated in 0.1 M PBS containing 500 μM AA, 30 μM DA, and 50 μM UA. Potential interfering species, including 1 mM glucose, fucose, sodium sulfate, potassium chloride, sodium chloride, and creatinine, were introduced. As shown in Figure 10c,d, the oxidation peaks of the primary analytes remained distinct and well-defined despite the presence of these interferents. Quantitatively, the peak current deviations for MWCNT-GR/GCE were 18.9%, 20.1%, and 31.0% for AA, DA, and UA, respectively, whereas MWCNT-GR-XC72/GCE exhibited markedly lower deviations of 8.09%, 2.86%, and 3.16%. This substantial reduction in current variation confirms the superior selectivity of the ternary carbon composite electrode. In summary, MWCNT-GR-XC72/GCE demonstrates clear advantages over MWCNT-GR/GCE across all evaluated metrics—including reproducibility, repeatability, stability, and selectivity—making it a highly promising platform for simultaneous electrochemical detection in complex matrices.

### 3.8. Analytical Application of MWCNT-GR-XC72/GCE in Real Samples

To evaluate the practical applicability of the developed sensor, human urine was selected as a real physiological matrix. The urine samples were diluted 50-fold with 0.1 M PBS to minimize matrix effects. Known concentrations of AA, DA, and UA were then spiked into the diluted samples, and the electrochemical responses were measured using DPV via the standard addition method, accounting for the endogenous uric acid background. The recovery was calculated using the following equation:

(5) Recovery (%) = Found (μM)/Spiked concentration (μM) × 100%

As summarized in Table 3, the MWCNT-GR-XC72/GCE effectively detected AA, DA, and UA across the tested concentration ranges, with recovery rates between 90% and 125%. At the lowest concentrations, recovery values were higher than 100% (up to 125% for UA), likely due to the proximity of these concentrations to the electrode’s LOD. This suggests that minor contributions from other electroactive species in the complex urine matrix that oxidize at similar potentials may also influence the current response. Therefore, future studies could benefit from optimized sample pretreatment to remove such interferences. Nevertheless, at spiked concentrations above the LOD, the sensor achieved excellent recoveries for the simultaneous detection of AA, DA, and UA. These results confirm that the proposed MWCNT-GR-XC72/GCE platform is capable of detecting these biomarkers in real physiological samples with high reliability, particularly at moderate to high concentrations.

## 4. Conclusions

In summary, a highly efficient electrochemical sensing platform was successfully developed for the simultaneous detection of AA, DA, and UA. This was achieved by modifying a GCE with a ternary carbon composite composed of MWCNTs, GR, and XC-72, prepared via a simple mixing method. The introduction of XC-72 into the MWCNT-GR matrix produced a synergistic effect, significantly increasing the electroactive surface area and providing abundant defect-driven catalytic sites, thereby enhancing electron transfer kinetics at the electrode interface. Consequently, the MWCNT-GR-XC72/GCE exhibited excellent analytical performance, with high sensitivities of 0.044, 0.47, and 0.95 μA μM^−1^ across the linear ranges of 100–1000 μM, 5–50 μM, and 10–100 μM for AA, DA, and UA, respectively. Furthermore, the sensor displayed low limits of detection (34.1, 4.23, and 11.1 μM) along with outstanding repeatability, reproducibility, and selectivity. The practical applicability of the sensor was confirmed by reliable simultaneous detection of these analytes in real human urine samples, yielding satisfactory recovery rates. These results demonstrate the significant potential of this cost-effective ternary carbon composite sensor for clinical diagnostics and physiological health monitoring.

## Figures and Tables

**Table 1:** Oxidation potential, Sensitivity, LOD and R^2^ of different electrodes for simulation determination of AA, DA and UA.

Electrode	Analyte	Oxidation Potential (V vs. Ag/AgCl)	Sensitivity (μA/μM)	LOD (μM)	R^2^
MWCNT/GCE	AA	−0.10	0.035	12.6	0.999
	DA	0.14	0.31	5.10	0.980
	UA	0.28	0.52	12.6	0.970
GR/GCE	AA	−0.06	0.029	94.2	0.983
	DA	0.16	0.54	3.75	0.989
	UA	0.30	0.86	73.6	0.990
XC72/GCE	AA	−0.08	0.024	68.6	0.991
	DA	0.15	0.44	5.36	0.979
	UA	0.29	0.42	12.15	0.973
MWCNT-GR/GCE	AA	−0.10	0.018	34.1	0.998
	DA	0.14	0.15	7.80	0.996
	UA	0.29	0.47	6.93	0.991
MWCNT-GR-XC72/GCE	AA	−0.08	0.044	34.1	0.998
	DA	0.15	0.47	4.23	0.987
	UA	0.30	0.95	11.1	0.977

**Table 2:** Comparison of reported electrochemical sensors and the present MWCNT-GR-XC72/GCE sensor for AA, DA, and UA detection.

Electrode	Linear Range (μM)			Detection Limit (μM)			Ref.
	AA	DA	UA	AA	DA	UA	
C_3_N_4_–GO/GCE	30–3000	0.25–320	2.5–1100	3.7	0.07	0.43	[3]
CB-CNT/PI/GCE	4 × 10^3^–2.6 × 10^4^	5–350	30–700	75	60	8.8	[8]
AuNPs-MoS_2_/GCE	10^3^–7 × 10^4^	0.05–4 × 10^3^	10–7 × 10^3^	100	0.05	10	[20]
RGO/GCE	0.1–400	2–600	0.7–100	0.1	1	0.7	[39]
NMC/GCE	5–4500	0.005–35	0.5–3500	0.15	0.0016	0.15	[40]
PDY/MWCNTs/GCE	5–110	0.0007–2.5	0.1–7.5	0.0099	0.0007	0.0089	[41]
MWCNT-GR-XC72/GCE	100–1000	5–50	10–100	34.1	4.23	11.1	This work

**Table 3:** Determination of AA, DA, and UA in diluted human urine samples using MWCNT-GR-XC72/GCE.

Analyte	Background (μM)	Added (μM)	Found (μM)	Recovery (%)
AA	0	100	115	115
		500	481	96
		1000	939	94
DA	0	5	5.82	116
		25	23.2	90
		50	47.5	95
UA	34.1	10	46.6	125
		50	79.9	92
		100	142	108

## Data Availability

The original contributions presented in this study are included in the article. Further inquiries can be directed to the corresponding author.

## Abbreviations

The following abbreviations are used in this manuscript:

MWCNT	Multi-Walled Carbon Nanotube
GR	Graphene
XC72	Vulcan XC-72 Carbon Black
GCE	Glass Carbon Electrode
AA	Ascorbic Acid
DA	Dopamine
UA	Uric Acid
LOD	Limit of Detection
CV	Cyclic voltammetry
DPV	Differential Pulse Voltammetry
